# Early postoperative gain in activity levels of lower extremity sarcoma survivors positively affects long-term physical activity and performance

**DOI:** 10.1007/s00520-023-07644-3

**Published:** 2023-03-01

**Authors:** Kevin Döring, Anna Vanessa Hegelmaier, Carmen Trost, Christoph Krall, Reinhard Windhager, Gerhard Martin Hobusch

**Affiliations:** 1 Department of Orthopedics and Trauma Surgery, Comprehensive Cancer Canter Vienna, Medical University of Vienna, Waehringer Straße 18-20, 1090 Vienna, Austria; 2 Center for Medical Statistics, Informatics and Intelligent Systems (CeMSIIS), Medical University of Vienna, Vienna, Austria

**Keywords:** Sports-activity, Limb salvage, Malignant bone tumor, SF-36

## Abstract

**Purpose:**

Little is known about the effect of sports activity levels on health-related quality of life (HRQOL) in long-term survivors of lower-extremity sarcoma.

**Methods:**

Eighty-three long-term survivors of bone and soft tissue sarcoma of the lower extremities with a median follow-up of 14 (range: 5–35) years completed the University of California and Los Angeles (UCLA) activity scores before tumor resection, 1 year after surgery and at the latest follow-up, as well as a Short Form 36 (SF-36) health survey at the latest follow-up. Simple linear regression models as well as stepwise variable selection with Akaike information criterion (AIC) were undertaken.

**Results:**

The preoperative UCLA activity level (median: 9, range: 2–10) dropped to a median of 4 (range: 1–10) 1 year after surgery before increasing to a score of 6 (range: 2–10) 5 years after surgery. The long-term SF-36 physical health component summary score (PCS) was 49 (SD: 9), and the mental health component summary score (MCS) was 54 (SD: 7). A linear model with stepwise variable selection identified a negative correlation of PCS with age at surgery (estimate: –0.2; *p* = 0.02), UCLA score at the last follow-up (estimate: 1.4; *p* = 0.02) and UCLA score 1 year after surgery (estimate: 1.0; *p* = 0.02).

**Conclusion:**

As not only the final activity levels but also the status immediately after surgery affect the PCS, higher early activity levels should be a goal of modern rehabilitation after sarcoma treatment. Further studies are needed to weigh the potential postoperative risks of higher sport activity levels against the benefits described in this study.

**Level of evidence:** Level 4.

## Introduction

After treatment, many sarcoma survivors stay inactive and experience an increased risk for persistent impairment [1, 2]. Local morbidity as well as negative systemic therapy effects may contribute to these limitations and restrictions in activities of daily living [3, 4]. It is known that a sufficient amount of physical activity is crucial for the recovery of cancer patients, with potential benefits on muscular strength, aerobic fitness, anxiety and functional quality of life (QOL) [5–8]. Moreover, exercise guidelines have been defined for breast, prostate, colon and hematologic cancer groups [7, 9]. For patients with sarcoma of the lower extremities, similar exercise recommendations for 150 min of endurance training per week exist [10].

Previous case series of bone and soft-tissue sarcoma survivors reported moderate to high sports activity levels after years of rehabilitation, especially when patients were used to high sports activity levels before their oncologic life events [11, 12]. However, sports activity levels are an expression of individual habits and may not be solely taken as an overall outcome measure after sarcoma treatment, as healthy sarcoma survivors with good limb function might still not participate regularly in personally achievable sports activities. The degree of postoperative activity loss, not necessarily the sports activity level, may severely impact survivors after restricting oncologic life events [13].

In the literature, little is known about associations of sports activity with the health-related quality of life (HRQOL) of patients after sarcoma resection. Findings on this topic should be of value for treatment specialists to further emphasize and advocate high postoperative activity levels in their patients. Therefore, the aim of this study was to analyze whether pre- and postoperative sports activity levels influence long-term HRQOL in survivors of sarcoma of the lower extremities. The following questions were asked: (1) How high were pre- and postoperative University of California and Los Angeles activity scores (UCLA) and the Short Form 36 health surveys (SF-36) at last follow-up? (2) What were the associations of UCLA activity scores on the SF-36 surveys?

## Patients and Methods

### Patients

This study comprised 105 long-term survivors of bone and soft-tissue sarcoma of the lower extremities. Patients were pooled from four different sarcoma cohorts and invited for interview [11, 12, 14, 15]. Patients prospectively completed an assessment of UCLA activity scores and SF-36 questionnaires at the time of interview. Additionally, at the time of interview, UCLA activity scores were retrospectively assessed at set time points 1 year before sarcoma resection as well as 1 year, 3 years and 5 years after resection. Inclusion criteria were (1) limb salvage surgery with resection of bone or soft tissue sarcoma in the (2) lower extremity, conducted in the (3) orthopedic department of the Medical University of Vienna between (4) 1972 and 2009 with (5) a minimal follow-up of 5 years after primary resection. Patients were excluded when interviews were not completed and data from SF-36 questionnaires or UCLA scores were missing. Twenty-two patients did not complete SF-36 short form questionnaires and were thus excluded from the statistical analysis.

Of the 83 patients included, 46 were male and 37 were female. The median age of patients at the time of surgery was 20 (range: 2–60) years. In total, 75% (*n* = 62) were treated for bone sarcomata, of whom 50% (*n* = 31) received treatment for osteosarcoma and the other 50% (*n* = 31) for Ewing sarcoma. Twenty-five percent (*n* = 21) of the study population was treated for soft tissue sarcoma in the lower extremities. In surgery, 30 (36%) patients received a proximal-tibial and distal-femoral replacement, 29 (35%) patients had soft tissue resection only, 12 (14%) patients received a proximal femoral replacement, 5 (5%) patients had a fibular pro-tibia operation, 4 (5%) patients underwent fibula resection, and 3 (4%) patients received a pelvic prosthesis. Twenty-nine (35%) patients received radiotherapy, and 28 (34%) patients received chemotherapy (VAIA protocol: 11 patients, VIDE-VAI/VAC protocol: 8 patients, EVAIA protocol: 6 patients, VACA protocol: 3 patients). The median follow-up was 14 (range: 5–35) years.

### Outcome parameters

#### UCLA (University of California, Los Angeles) sports activity score

The UCLA assessment is based on a scale from 1 to 10 depending on patients’ physical ability. Level 1 shows a total physical inability and dependency on others, while level 10 is defined as a maximum of activity a person can reach (for example, regular participation in challenging sports such as tennis or skiing). The frequency or intensity of various sports is not taken into consideration [16, 17]. The UCLA assessment was performed prospectively at the time of the interview and retrospectively for different set time points (preoperative, 1, 3, 5 years postoperative).

#### SF-36 (36-Item Short Form Health Survey)

The 36-Item SF-36 was used to measure HRQOL, which is defined as patient factors related to physical and mental health. In this questionnaire, 8 subcategories are summed as the “physical health component summary score” (PCS, including scales of physical functioning, physical role, bodily pain and general health, with a total of 21 items) and the “mental health component summary score” (MCS, including scales of vitality, social functioning, emotional role and mental health, with a total of 14 items). Physical health was defined by the PCS, and mental health was defined by the MCS in this study [18]. The SF-36 was evaluated postoperatively once at the time of interview.

### Statistical analysis

Pairwise differences between preoperative values and values after 1 year and at the last follow-up were analyzed. Linear models for the MCS scale and PCS scale on sex, age at surgery, age at latest follow-up, and all UCLA scores were analyzed. Bonferroni correction of the *p* value for all effects on the main endpoint of MCS and PCS was performed. Naive *p* values are reported with respect to secondary endpoints. To disentangle the effect of the postoperative UCLA values from the differences to reported preoperative values, an interaction term between pre- and postoperative values was included in the regression models. Thereafter, a linear model with stepwise variable selection by AIC was performed with differences from preoperative values as well as between consecutive measurements. A *p* value < 0.05 was considered significant. Statistics were performed using *IBM SPSS Statistics 26* software (*International Business Machines Corporation*, *IBM*; Armonk, NY, USA).

## Results

### UCLA activity levels and SF-36 PCS and MCS summary scores

**Fig. 1:**
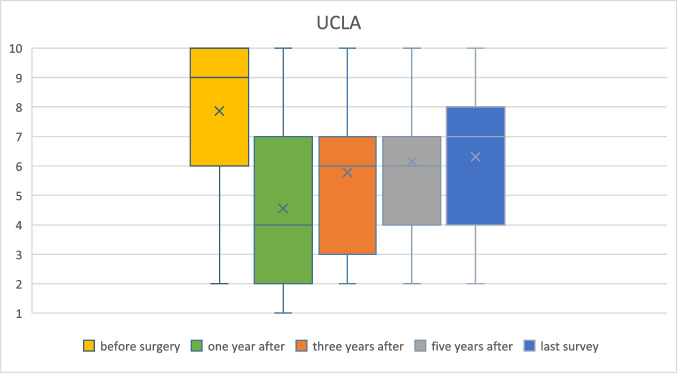
Boxplots of UCLA scores one year before surgery and one year, three years, and five years after surgery as well as at the last survey. “X” indicates the mean

The median preoperative UCLA activity level was 9 (range: 2–10). The median UCLA decreased from the preoperative value to the first value after surgery (median: 4, range: 1–10) before increasing to a median of 6 (range: 2–10) (Fig. 1) 5 years postoperatively. The mean value for MCS was 54 (SD: 7), and that for PCS was 49 (SD: 9) at the last follow-up.

### Associations of SF-36 scores with UCLA activity levels and their dependence on covariates

**Fig. 2:**
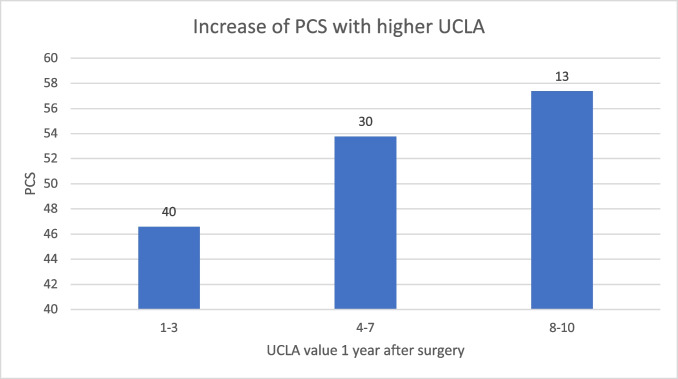
Illustration showing an increase in PCS at the last follow-up with a rising UCLA score one year after surgery (*p* = 0.02). Patient numbers are indicated above the columns

**Fig. 3:**
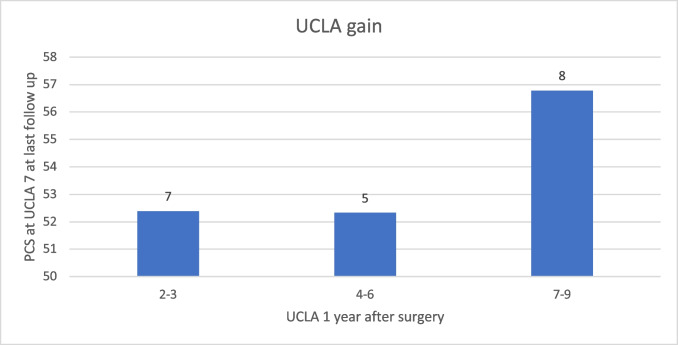
Example of UCLA score gain after surgery, with patients reporting a UCLA value of 7 at the last follow-up. The X-axis shows the UCLA score one year after surgery. Patients reported a higher PCS when the drop one year after surgery was less pronounced (*p* = 0.02). Patient numbers are indicated above the columns

Simple linear regression models identified a negative association of the PCS scale, but not the MCS scale, with age at surgery and positive correlations with UCLA measurements after surgery at all set time points 1, 3 and 5 years postoperatively. In statistical analysis, a positive association with differences between self-reported preoperative UCLA and all postoperative UCLA measurements, but not with differences between the first measurement after surgery and consecutive measurements, could be seen. No associations of PCS with sex, follow-up period or reconstruction methods were observed (Table 1). To disentangle the effects of the postoperative UCLA values on PCS from the differences between reported preoperative and postoperative UCLA values, an interaction term between pre- and postoperative values was included in the regression models. However, no significant effect of the interaction term on PCS could be identified, suggesting that the most influential factor that determines PCS is the current latest follow-up value of UCLA rather than the difference from the preoperative value. A linear model with stepwise variable selection with Akaike information criterion (AIC) identified negative correlations of PCS with age at surgery (Estimate: –0.2; *p* = 0.02), correlations with UCLA at last follow up (Estimate: 1.4; *p* = 0.02) and correlations with UCLA 1 year after surgery (Estimate: 1.0; *p* = 0.02, Fig. 2). This result underlines that not only the final value of UCLA but also the status immediately after surgery affects PCS. Therefore, patients with identical UCLA values at last follow-up will tend to report higher PCS if the low after surgery is less pronounced (Fig. 3). A linear model with stepwise variable selection including age at surgery and all differences between consecutive UCLA values by AIC identified a negative correlation with age at surgery and a positive correlation with the difference between the first postoperative UCLA value and the preoperative UCLA value (Estimate: 1.2; *p* = 0.002).

**Table 1:** Dependence of PSC and MSC on covariates

	PCS			MCS		
	Estimate	Std. error	Pr( >|t|	Estimate	Std. error	Pr( >|t|
Sex	− 1.3	2.2	0.5	− 0.6	1.7	0.7
Age at surgery	− 0.2	0.1	**0.04**	0.06	0.07	0.4
Follow up time	− 0.08	0.2	0.6	− 0.2	0.1	0.3
UCLA before surgery	0.2	0.5	0.6	− 0.2	0.4	0.7
UCLA one year after surgery	1.4	0.4	**0.001**	0.5	0.3	0.1
UCLA three years after surgery	1.4	0.4	**0.001**	0.07	0.4	0.8
UCLA five years after surgery	1.6	0.5	**0.001**	0.5	0.4	0.2
UCLA last survey	1.8	0.5	**0.001**	0.5	0.4	0.2
Reconstruction	− 0.9	2.2	0.7	3.6	1.7	**0.04**
Difference UCLA before surgery to one year after surgery	1.2	0.4	**0.002**	0.6	0.3	0.08
Difference UCLA before surgery to three years after surgery	1.3	0.4	**0.003**	0.2	0.4	0.6
Difference UCLA before surgery to five years after surgery	1.4	0.5	**0.006**	0.7	0.4	0.1
Difference UCLA before surgery to last follow-up	1.5	0.5	**0.002**	0.6	0.4	0.1

The level of significance is p<0.05

## Discussion

Patients with bone and soft tissue sarcoma of the extremities particularly suffer from major postoperative physical constraints inherent to tumor resections. These limitations may lead to lasting burdens on professional and personal life [3, 19]. Therefore, a deeper understanding of connections between physical ability and HRQOL after extremity sarcoma resection is mandatory for the promotion of convalescence through rapid and standardized physical rehabilitation programs. We found that sarcoma resection had permanent effects on the physical ability of our patient population, as the patients were not able to achieve their preoperative UCLA activity levels after surgery. We further found that early postoperative activity levels influenced long-term physical activity and performance. This information underlines the importance of early postoperative rehabilitation protocols and may further advocate physical exercise in this sensitive time frame with careful attention to patient-specific functional limitations (Table 2).

**Table 2:** A linear model with stepwise variable selection by AIC identified a negative correlation with age at surgery and a positive correlation with UCLA 1 year after surgery and UCLA at the latest follow-up

	Estimate	Std. error	*T* value	*P*
Age at surgery	− 0.2	0.1	− 2.4	**0.02**
Follow up time	− 0.3	0.2	− 1.8	0.07
UCLA before surgery	− 0.8	0.5	− 1.7	0.1
UCLA one year after surgery	1.0	0.4	**2.3**	**0.02**
UCLA last survey	1.4	0.6	**2.3**	**0.02**

The level of significance is p<0.05

### Limitations

By including patients treated with limb-salvage sarcoma surgery of the lower extremities between 1972 and 2009, a period spanning three decades was chosen. Because of this time frame, both surgical techniques and postoperative mobilization schemata changed over time. It must be assumed that subsequent patients had advantages in postoperative mobilization due to the progression of physiotherapeutic regimens. An inclusion of patients treated in the last century adds even further relevancy to this study, as modern physiotherapy and easier access to sport institutions might have substantial long-term benefits on potential postoperative rehabilitation and thus physical performance. In this study, no discrimination between different resection depths or compartments was undertaken, and thus no associations of sports activity and HRQOL on different localizations of the lower extremity could be analyzed. It must be assumed that patients with shallow sarcoma had advantages in postoperative mobilization due to fewer functional limitations and fewer restrictions of postoperative rehabilitation guidelines. Thus, a high early postoperative activity level should be understood as a multimodal result of successful rehabilitation protocols, high patient motivation, and fewer invading tumors. Conversely, a limited potential UCLA activity score needs to be assumed in patients with high surgical morbidity.

### Postoperative decline in physical ability in long-term sarcoma survivors

In line with similar studies, this study showed that the postoperative UCLA sport activity scales never again reached preoperative values. Research on the positive effects of physical activity with respect to psychological and physical parameters in cancer patients and survivors has steadily gained importance over the last few years [20, 21]. However, only 20–40% of cancer patients reach the recommended amount of daily physical activity [22]. Huy et al. demonstrated that physical activity sharply decreases during chemo- and radiotherapy, while participation in rehabilitation programs was associated with an increase in physical activity even after therapy [23]. With the additional information that physical therapy improves many aspects of everyday life, such as cancer fatigue, muscular strength, anxiety and self-esteem, it is evident that participation in physical therapy and rehabilitation programs should be mandatory for cancer patients and survivors [6, 24–26]. This might be even more true for sarcoma patients after tumor resection in comparison to the general cancer population, as extremity tumors might lead to a more severe functional impairment due to localization and thus come with a higher scope for improvement and adjustment in physical therapy. A tendency toward active remobilization in cancer patient treatment can be observed internationally. However, there are only a few defined recommendations reporting on the efficacy of exercise on specific outcomes, such as physical function, fitness and QOL, in oncologic patients [9, 27, 28]. To ensure the maximum treatment effect, endurance and resistance training should always be personalized to patients’ sex, age and current therapy modality, as women, young patients and patients receiving platin-based chemotherapies are at a higher risk for physical activity decline [10]. Further studies comparing defined rehabilitation protocols both during the adjuvant treatment period and in long follow-ups are needed to provide the highest benefit for cancer patients, especially in patients surviving extremity sarcoma.

### Associations of UCLA activity levels on the SF-36

This study showed that differences in pre- and postoperative UCLA sports activity scores resulted in a reduction of the SF-36 PCS and thus a decrease in patients’ HRQOL. As the UCLA sports activity level 1 year after surgery had associations with SF-36 PCS scores at the latest follow-up, the importance of early postoperative rehabilitation is shown. To draw further conclusions from these results, the best possible postoperative physical therapy would result in a less prominent decline in physical activities at an early postoperative stage. This result is of value for treatment specialists, as patients should be informed about the importance of early mobilization. Patients should be strictly advised to participate in rehabilitation programs and physiotherapy during the year after surgery to increase their physical capabilities and HRQOL even in the long run. In cases of extensive resections or limitations due to reconstructive procedures, such as implantation of modular megaprostheses or reconstruction of nerves and vessels, physical activity and sports should be closely monitored and allowed in safe intervals with potential postoperative risks weighted against benefits regarding HRQOL and physical performance described in this study. Raising awareness of physical limitations after cancer treatment is crucial, as bodily constraints can lead to massive burdens in professional as well as personal life and therefore have a huge impact on everyday life even years after primary surgery [3]. Several meta-analyses have reported on physical activity and its positive influence on HRQOL in cancer survivors [6, 28–31]. This current study shows similar relations between activity levels and SF-36 PCS in survivors of lower-extremity sarcoma. Thus, we recommend high postoperative activity levels to optimize mobilization and long-term physical performance.

## Conclusion

The postoperative activity levels of survivors of lower-extremity sarcoma did not reach preoperative values. In conjunction, the early postoperative physical level reflects higher long-term physical activity and performance in these patients. This information emphasizes the importance of customized rehabilitation for the efficacy and safety of mobilization protocols. Further studies and guidelines are needed to optimize rehabilitation in this sensitive timeframe, especially in patients receiving extended resections and reconstructions due to extremity sarcoma.

## Data Availability

The datasets used for this study are available from the corresponding author on reasonable request.
